# Implementing psychosocial guidelines into specialized spinal cord injury rehabilitation services to strengthen person-centred health care: protocol for a mixed methods study

**DOI:** 10.3389/fresc.2025.1537890

**Published:** 2025-04-07

**Authors:** Candice McBain, Anne Marie Sarandrea, Ilaria Pozzato, Mohit Arora, Daniel Myles, John Bourke, Yvonne Tran, Ian D. Cameron, James W. Middleton, Ashley Craig

**Affiliations:** ^1^ John Walsh Centre for Rehabilitation Research, Northern Sydney Local Health District, Sydney, NSW, Australia; ^2^ Kolling Institute, Faculty of Medicine and Health, The University of Sydney, Sydney, NSW, Australia; ^3^ Spinal Cord Injury Unit, Royal North Shore Hospital, Northern Sydney Local Health District, Sydney, NSW, Australia; ^4^ NSW State Spinal Cord Injury Service, Agency for Clinical Innovation, Sydney, NSW, Australia; ^5^ Macquarie University Hearing, Faculty of Medicine, Health and Human Sciences, Macquarie University, Sydney, NSW, Australia

**Keywords:** psychosocial guidelines, adjustment, implementation, patient-centred care, behaviour change techniques, spinal cord injury, facilitators, barriers

## Abstract

**Background:**

Spinal cord injury (SCI) is a severe neurological disorder resulting in loss of movement and altered sensation with lifelong impacts on health, function, and social integration. Multidisciplinary SCI rehabilitation primarily focuses on enhancing function and independence while simultaneously managing secondary health conditions and providing psychosocial support. Therefore, a major goal in SCI rehabilitation should be strengthening patients’ capacity to cope with and adjust to challenges they encounter. Using a mixed methods design, the primary aim of this study is to integrate psychosocial guidelines that promote psychological adjustment into SCI rehabilitation, and second, to evaluate facilitators and barriers to their successful implementation.

**Methods:**

To determine perceived depth of knowledge, beliefs, and attitudes about psychosocial care, and usage of psychosocial guidelines, healthcare professionals in the three specialist SCI services in New South Wales, Australia will be invited to complete a baseline survey. Following the survey, semi-structured one-to-one interviews and focus groups will be conducted with healthcare professionals representing different health disciplines to understand the context and generate ideas about how best to integrate these guidelines into clinical practice. Based on the surveys, interviews, and focus groups, an implementation intervention employing educational strategies, structural, and nudge (behavioural change) approaches will be designed and implemented over a period of 18-months to facilitate integration of the guidelines into the SCI services. A post-intervention survey with healthcare workers will then be conducted. Focus groups from each SCI service, with representation across the different healthcare professions, will also be conducted to identify facilitators and barriers to implementing the guidelines. Success of implementation will be determined by analyzing any shifts in perceived knowledge, attitudes, and behaviour of staff and cultural/structural processes observed through comparing baseline and post-intervention qualitative and quantitative data. To capture lived experience insight, 10 patients with SCI currently undergoing rehabilitation will be interviewed.

**Discussion:**

This study will establish the success of implementing psychosocial guidelines into three specialist SCI services. It is hypothesized that constructive changes will occur in the knowledge, attitudes, and behaviour of the SCI Unit healthcare professionals, leading to improved psychosocial practices and patient outcomes that will strengthen person-centred healthcare in SCI rehabilitation. This study has been retrospectively registered with the Australian New Zealand Clinical Trials Registry on the 7th of May 2024. The registration number is: ACTRN12624000581561

## Background

Spinal cord injury (SCI) is a severe injury to the spinal cord resulting from either a traumatic or non-traumatic cause that is associated with disability (1, 2). In the past, SCI has been more prevalent among males, particularly those aged 16–30 years of age due to a higher risk of traumatic injury, such as a road traffic crash or sporting injury (3). More recently, there has been a further demographic shift towards a bimodal age distribution whereby countries with ageing populations, like Europe, the USA and Australia, have seen an increase in the rate of SCI in older people, particularly females, aged 65 years and older sustaining a SCI following a fall (3, 4). Thus, we now see two distinct groups within the traumatic SCI population, those injured in early adulthood and those injured in older age. Additionally, non-traumatic, non-progressive spinal cord conditions can result from illness or disorders, such as spinal stenosis, transverse myelitis, epidural abscess, spinal cord ischemia, or hemorrhage (5).

SCI is often associated with substantial physical disability and likelihood of secondary health conditions, such as chronic pain, spasticity, autonomic dysreflexia, cardiovascular disease, pressure injuries, respiratory complications, and urinary and bowel problems (6). Psychological comorbidities including depressed mood and anxiety disorders, as well as catastrophizing styles of thinking are also prevalent in adults with SCI (7–9). Thus, alongside inpatient medical treatment and rehabilitation, accurate evaluation, diagnosis, and treatment of mental health conditions is important since psychological and emotional distress is common following SCI (7–9) and may contribute to these common secondary health conditions.

People with a SCI are at greater risk of developing mood and anxiety disorders, suicidal thoughts, and self-perceived stress compared with the general population (7–9). Yet secondary mental health conditions often go undiagnosed, particularly during the early stages post-injury when the primary focus is on improving the physical function of people with SCI. Psychological comorbidities are clinically relevant and require clinical attention as they can interfere with the intensive learning process involved in rehabilitation following SCI, especially when they go undiagnosed (2). For example, if depression is undiagnosed and left untreated, it may have a longer duration and become harder to treat over time (10, 11), In turn, secondary psychological comorbidities that go untreated may impede adjustment to the community following discharge from rehabilitation, potentially hindering return to work and social participation (2, 7, 9), and may lead to additional psychosocial disability for people living with SCI.

Cognitive impairment is another common co-occurring condition after a SCI (12–15), with a pre-morbid history or comorbid brain injury being a frequent contributor (12, 13, 15). In fact, it was recently found that the increased likelihood for an adult with SCI to have cognitive impairment was 18 times higher than for an able-bodied adult (16). Despite this, it is concerning that a brain injury, especially minor traumatic brain injury (mTBI), can often remain undiagnosed and thus untreated (17, 18). This can hinder the uptake of self-managed behaviour and the development of independent living skills (17–19), ultimately disrupting the overall process of rehabilitation and adjustment to SCI. Yet even in the absence of a concomitant mTBI, it is not uncommon for adults of all ages with a SCI to experience impairments in their cognitive functioning due to a range of factors like cardiac regulation problems, fatigue, psychological disorder, chronic pain, older age, sleep disorder, and neural inflammation (12–17, 19, 20). For example, fatigue has been found to be associated with delayed memory performance and experiencing pain can reduce attention capacity (20, 21). Additionally, recent research found that mental health issues such as elevated anxiety are linked to a decline in working memory (21). Considering that mental health conditions like anxiety can often be undetected during SCI rehabilitation, there exists an inherent risk of negative adjustment to the injury (2, 21). A further complication to the adjustment process is that up to 56% of people with a SCI have been found to meet the common criteria for polypharmacy, defined as at least five medications prescribed concomitantly, which can also be associated with cognitive impairment (22). More so polypharmacy is of particular concern for people with advancing age (23) due to the risk for delirium (24).

Employing a biopsychosocial model of care for SCI rehabilitation is key to delivery of person-centred care (2). This model encourages healthcare professionals to consider physiological, psychological, and social contributors to a person's health care experience (25, 26). In addition to guidelines that address the physical aspects of rehabilitation for individuals with SCI, the authors contend there is a need to integrate psychosocial guidelines into the rehabilitation process. Psychosocial guidelines aim to provide healthcare professionals with essential insights into how to communicate with, support, and engage/motivate patients with a newly acquired SCI during their rehabilitation, especially when cognitive impairment is present (2, 26). Such a holistic approach seeks to optimize person-centred care for these individuals. Healthcare professionals are in a unique position to provide not only physical health care, but also psychosocial health care to inpatients with a SCI in the first months after their injury. The value of integrating psychosocial care guidelines and employing patient-centred goals into the SCI rehabilitation setting will, arguably, positively influence how patients adjust to their injury (2, 26, 27). However, many healthcare professionals working in SCI acute care and rehabilitation settings often lack the necessary training to apply psychosocial care as part of standard practice for everyone (26). For example, healthcare professionals working in the acute and rehabilitation phases need to have the confidence to screen for symptoms of mild cognitive impairment and mental health comorbidities in their patients, and in response, have the confidence to integrate and adopt psychosocial care strategies to foster optimal coping and adjustment skills in their patients (26).

A Cochrane review suggested the uptake of clinical practice guidelines in areas such as cardiology and cancer care can be improved if implementation tools encourage the adoption, penetration, and sustainment of the guidelines into routine practice by healthcare professionals (28). In the USA, professional standards of practice have been developed for SCI rehabilitation staff (29), though we are unaware of any evidence about the success of the integration of these standards into practice. In the United Kingdom (UK), a psychological care pathway model and best practice guidelines are being developed, drawing upon guidelines being used by the National Spinal Injuries Centre Stoke Mandeville Clinical Psychology Service (30), however, again, there is no evidence yet available of the success of their integration into SCI services in the UK. Clinical practice guidelines for managing mental disorders after SCI have also been developed by the Paralyzed Veterans of America Consortium for Spinal Cord Medicine, yet no evidence is available for their implementation (31).

There is a growing body of research on various methods for supporting clinician decision-making to align more closely with using specific evidence-based practices (32), with distinct strategies that can be categorized as passive (such as posters) or active (such as workshops) (32, 33). A novel way to strengthen clinical practice in line with desired standards is the “nudge” paradigm for behaviour change (32, 34). The premise is that behaviour can be voluntarily shifted by making specific choices instinctively appealing (35). Furthermore, an enabling culture that supports clinicians to engage in the process of change is also recognized as important for successful integration of new standards of practice (36).

In Australia, a Psychosocial Guide was developed for healthcare staff working in SCI rehabilitation but was never thoroughly integrated into the SCI rehabilitation process (37). Consequently, in a strategy to integrate this Guide into practice more effectively, it was recently upgraded by researchers and representatives from the multidisciplinary team of healthcare professionals working in SCI rehabilitation, as well as a colleague with lived experience of SCI (38). During this developmental and consultation stage in which a substantial upgrade of the prior 2014 psychosocial guide designed for SCI rehabilitation was completed, evidence regarding relevant psychosocial practice guidelines was accumulated by conducting a series of literature reviews over a 6-month period using primary search terms such as “*spinal cord injury*”, “*rehabilitation*”, “*adjustment*”, “*treatment*”, used in combination with secondary search terms such as “*depression*”, “*pain*”, “*cognitive impairment*”, and “*social participation*” (39)*.* When the guide was updated, consultation occurred by subjecting these guidelines to review by a diverse range of expert healthcare professionals involved in the medical and psychosocial care in specialist SCI services in NSW, Australia. Consensus was also sought for a new section on cognitive impairment in patients with SCI participating in rehabilitation, designed to be inserted into these guidelines. This was eventually achieved by conducting a rigorous e-Delphi study with the provision of evidence-based statements. Participants in the e-Delphi study were all experts in SCI rehabilitation, including one member with lived experience of SCI. These cognitive impairment guidelines were integrated into the updated guide when consensus was reached. Thus, a major upgrade to the recent psychosocial guide were strategies for the psychosocial management of SCI patients with cognitive impairment. The updated Guide is now publicly available online (38, 39) and was developed to support multidisciplinary healthcare professionals to improve their person-centred care of patients with acute SCI.

### Aims and objectives

Recognizing the difficulties of integrating past versions of the Psychosocial Guide into SCI rehabilitation practice, the primary aim of the intervention is to implement and integrate the latest Guide (upgraded in July 2023) into the three specialist SCI services in NSW, Australia. To facilitate this, a mixed methods approach will be employed to develop a multi-dimensional implementation intervention based on systems thinking (e.g., players, processes, interactions, technology) and focussed behavioural change techniques. The implementation objectives involves the use of the Consolidated Framework for Implementation Research (CFIR) (40), providing an ideal structure with which to explore individual, team, and organizational (inner setting) and health system level (outer setting) barriers and facilitators to the implementation of the Guide. This will be achieved through interviews and focus groups with the multidisciplinary healthcare professionals in SCI services and individuals living with SCI, according to CFIR domains. Further to this, the implementation objectives are to increase awareness, knowledge, and understanding of the Psychosocial Guide, and to co-design a set of implementation strategies and embed a plan to promote sustainability of the intervention in the SCI services.

## Methods/design

### Settings

The study will be conducted in three specialist SCI services in NSW, Australia, with one facility mostly providing acute care, another only subacute rehabilitation and a third Unit providing both acute care and subacute rehabilitation. The three SCI services have variations in catchment areas, as well as infrastructure and organisation of professional teams, workflows, and processes for SCI rehabilitation.

### Participants

Healthcare professionals including nurses, physiotherapists, occupational therapists, speech pathologists, medical specialists, social workers, psychologists, and psychiatrists working in the three SCI services will be invited to participate in an online survey pre-and-post intervention that will determine their understanding and awareness of psychosocial SCI care principles and practices. A selection, based on expertise and availability, of these participants from the different disciplines (*n* = 15) will then be invited to attend one-on-one interviews that will inform a tailored set of strategies to support changing or strengthening clinician behaviours in adopting the evidence-based guidelines. After the intervention, a further selection of healthcare professionals will again be invited to participate in either an interview or a focus group (again depending on their availability) to determine facilitators and barriers to implementation. Inclusion criteria will consist of (a) healthcare professionals from each of the three SCI services located at three NSW hospitals who volunteer to participate in the study; (b) a minimum of 12-months work experience; (c) representation by all SCI disciplines, and (d) being able to communicate effectively in English. The researchers will invite key informants that meet the inclusion criteria to be interviewed to ensure we capture the views of a broad range of healthcare professionals.

Since the major aim of this project is to introduce the Guide for the first time to the three SCI services in NSW, in order to encourage adoption, penetration, and sustainment into practice, given the nature of this research we believe there is no value for using a control/comparison group in this project.

Additionally, up to 10 adults aged between 18 and 80 years with SCI, able to communicate effectively in English, who are currently undergoing rehabilitation, and not experiencing severe mental illness or at risk-behaviours will also be interviewed, to obtain their perceptions and awareness of psychosocial care during rehabilitation. Patients with SCI who meet the inclusion criteria will be invited to participate. They will be interviewed at one of the three sites in-person by a member of the research team.

### Mode of assessment

(a) Healthcare professionals

The online survey will be administered pre-and-post intervention to healthcare professionals currently employed at one of the three participating SCI services (*n* = 60). The online survey will request minimal socio-demographic information such as age range, sex, employment status, workplace, type of employment, years working and health discipline area. Forced choice questions will explore healthcare professionals perceived knowledge about psychosocial care and the Psychosocial Guide, person-centred care, beliefs about person/patient care, and attitudes towards psychosocial care. The survey questions developed by the researchers aim to develop an understanding of the beliefs and values of the healthcare professionals as part of the contextual factors of the CFIR. No validated scales are being used. The data will be stored on a secure online platform called Research Electronic Data Capture (REDCap) (41).

The semi-structured one-to-one interviews will take place pre-and-post intervention with up to 15 healthcare professionals via teleconference (i.e., via Zoom). Baseline interviews will provide information in relation to healthcare professionals understanding and awareness of psychosocial care principles, current culture and practices, identify barriers and elicit potential nudge and other implementation strategies for an intervention to increase understanding of the guidelines and enhance psychosocial care practice. Follow-up interviews will capture information about the impact of the intervention on the implementation of the Guide in the SCI services. Open-ended questions will explore issues related to perceived competence, knowledge, and skills related to psychosocial care, as well as perceived facilitators and barriers to implementation of the Guide in the SCI Unit, such as culture/social norms, compatibility, relative priority, readiness to change, training, and available resources. For example, interviews will explore questions such as ‘*Are there any barriers or challenges that you foresee in implementing these guidelines in your SCI unit*?’ and ‘*What do you think would make the Guide more easily accessible to you and other multidisciplinary staff*?’.

Interviews will be audio recorded with the participant's permission and transcribed verbatim by a University of Sydney approved transcription service. All participants will be de-identified and given a participant number while all data will be re-identifiable and stored on a University of Sydney approved secure online server (i.e., REDCap).

Pre-and-post intervention focus groups with healthcare professionals (*N* = 20) will provide feedback and their views about psychosocial care, the current utility of the Guide in SCI services, and post-intervention on the impact of the intervention on the implementation of the guidelines in the SCI services. Open-ended questions will explore issues related to perceived knowledge about and attitudes towards psychosocial care, current practice and changes in clinical practice as well as facilitators and/or barriers to implementation of the guidelines in the SCI Unit. Participation in the focus groups will be confidential. Focus groups will explore questions such as ‘*If you suspect someone has cognitive impairment, what do you do*?’ and ‘*What strategies or facilitators do you think would make the implementation of new guidelines in the SCI services more effective*?’.

Focus groups will be audio recorded with the participant's permission and transcribed verbatim by the approved transcription service. Focus groups will be conducted face-to face or via teleconference (i.e., on Zoom) at one of the three participating SCI services.

(b) Adults with SCI

The semi-structured interviews with patients with SCI who have undergone SCI rehabilitation at one of the participating SCI services (*n* = 10) will involve them reflecting on their inpatient rehabilitation experience as a recipient of psychosocial care. Open-ended questions will explore issues related to expectations, perceived needs, and satisfaction of care through questions such as ‘*Were you involved as much as you wanted to be in decisions about your care and treatment*?’ and ‘*Can you tell me in what way your emotional needs have or have not been met during your hospital stay*?’.

The feedback from the patients with SCI (*n* = 10) will help to determine the current attitudes and behaviours of the healthcare professionals through the eyes of the patients and also give an indication of how the psychosocial needs of patients are being met through patient-staff interactions. This will assist the researchers in deciding which section of the Guide to focus on during the implementation intervention.

### Study procedure

**Figure 1:**
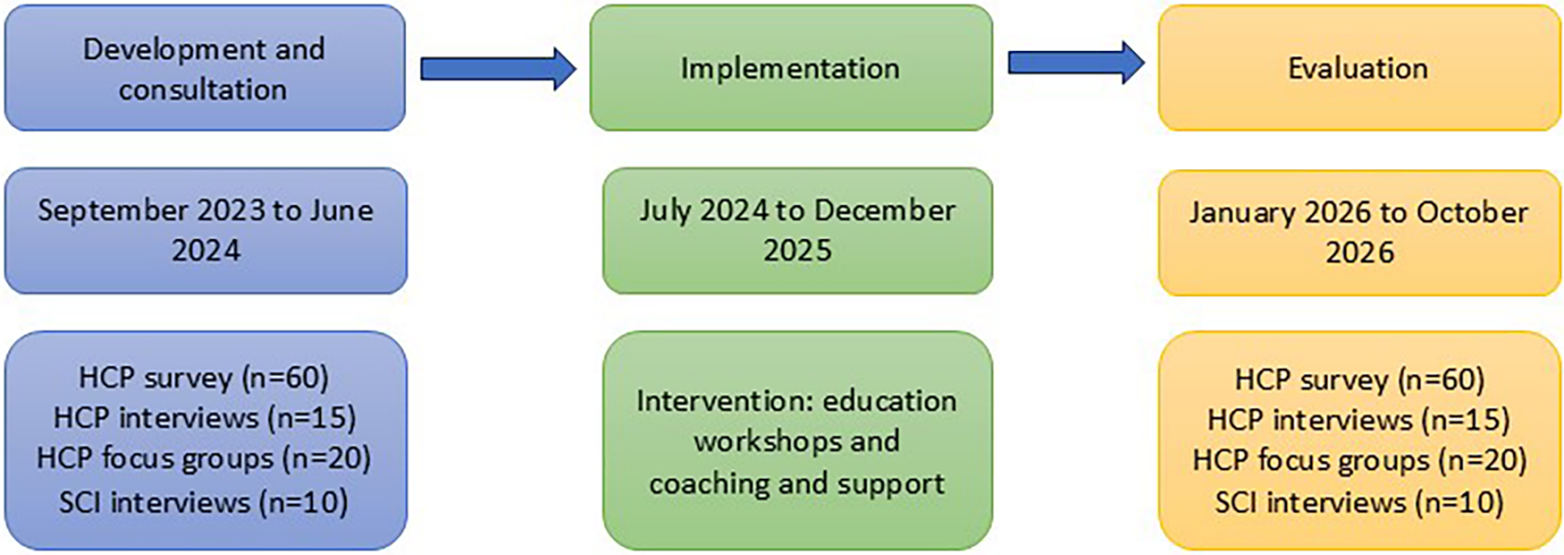
Study procedure.

The study involves three stages as illustrated in Figure 1.

### Stage 1: Development and consultation

In Stage 1, we will assess the context for implementation through surveying a range of healthcare professionals to gauge the organisational commitment and readiness within the services to implement a more systematic application of the psychosocial guidelines. Interviews and focus groups with healthcare professionals will advise on the current climate of the services, engage key healthcare professionals to help promote guideline uptake and adherence, and help to elicit potential strategies for the implementation intervention. The baseline interviews with patients with a SCI (*n* = 10) will document experiences of psychosocial care delivery in the three SCI services. Themes and information derived from Stage 1 will guide the development of a range of active implementation strategies used to aid implementation in Stage 2 that will help to embed the Psychosocial Guide into clinical practice and institutionalize cultural changes.

### Stage 2: Implementation

The reporting of this study was conducted in line with the Standards for Reporting Implementation Studies (StaRI) checklist (42). This stage consists of a multi-dimensional implementation intervention that will be conducted over a period of 18-months. Findings from the baseline qualitative (interviews and focus groups) and quantitative (surveys) data will be mapped to the organizational (inner setting) and health system level (outer setting) CFIR domains (40, 43). This process will enable the identification of any potential barriers and facilitators (e.g., communications, culture, tension for change, compatibility, readiness, resources, partnerships, policies, societal pressure and so on) that may influence implementation effectiveness within the SCI Unit setting from both an organisational, and systems perspective, respectively. These barriers and facilitators will be considered when determining the most appropriate mode of delivery for the intervention as well as methods to embed the use of the Guide in the SCI services post-intervention to promote sustainability.

Based on these findings we will seek to reshape the underlying ‘choice architecture’ (i.e., the interpersonal and relational environment in which clinical decisions are made), by designing different ways in which choices can be presented to individuals (33, 44, 45), including modifying the social and physical environment to enhance the capacity for clinicians to generate behaviours (e.g., to overcome unconscious biases) to improve the decision-making process. Such approaches will include various nudge and other enabling strategies (i.e., education sessions, information transparency, peer comparison, active choice, alerts and reminders, or environmental cueing/priming) (46), that may positively influence decision-making, behavioural changes in healthcare professionals, and structuring of processes and actions within the individual and multidisciplinary organisation and systems on the SCI services. The purpose of such an implementation intervention being to enhance clinical practice in line with the 2023 version of the Psychosocial Guide. We will utilize this range of behaviour change and nudge strategies with the understanding that healthcare professionals can be influenced in the desired direction regarding both uptake and adherence to the new guidelines, provided they are committed to implementing psychosocial care and see the Guide as conducive to facilitating psychosocial care. Healthcare professionals’ knowledge, attitudes, and capability around psychosocial care are believed to be major drivers for guideline adherence. As such, educational toolkits, training videos, and structured workshops will be designed (see Supplementary Table S1). This approach will be utilized through conducting professional discipline-specific education and training sessions with targeted strategies designed for each discipline. For example, nursing staff will encounter different psychosocial challenges to allied health professionals, so strategies used in the intervention will need to be tailored to the staff. An adjunct strategy will be to build and foster a guiding coalition (community of practice within the unit) with designated “champions”, who can model, support and influence staff behaviours.

Lack of awareness is thought to be another barrier, thus a “priming” nudge, which is the provision of cues in the environment (e.g., infographic posters) and the use of reminders (e.g., emails) is also a feasible strategy. For example, psychosocial stories, feedback from patients, and case studies can be regularly disseminated through email as well as discussed in regular team meetings. These nudge methods have an evidence base as confirmed in a recent systematic scoping review, in which they were examined for their utility and efficacy (35).

Thus, the implementation tools designed by the researchers will include a “nudge” paradigm (e.g., educational toolkit, training videos, infographics, screen savers) to promote the successful adoption, penetration, and sustainment of the Guide in the SCI services post-intervention (see Supplementary Table S1 for more detail).

It is hypothesized that the implementation intervention will promote the successful adoption, penetration, and sustainment of the Psychosocial Guide in the SCI services through the intervention strategies aimed at influencing domains like “inner settings” (e.g., unit culture/social norms, compatibility with existing work structures, learning climate, engagement of leadership, readiness for change and implementation, communication networks). A variety of “time sensitive” interventions will be trialled, such as education sessions designed for each discipline (e.g., regular team/discipline-based seminars, employee onboarding education sessions, discussions of team cultures), and training skills-based workshops designed to encourage the use of psychosocial skills in accord with the guidelines (e.g., audit and feedback, case studies, and active choice through prompts).

The researchers will develop educational material to be presented to the healthcare professionals through in-person workshops at the SCI services. The workshop will incorporate a PowerPoint presentation as a key instructional tool to enhance the delivery of educational content. This material will be designed to introduce the Guide to the healthcare team and provide a basic overview of the content and purpose of the Guide. A practical workshop will also be delivered by the researchers in-person at the SCI services that will provide an opportunity for the healthcare professionals to apply the Guide to clinical case studies that are reflective of patients they typically work with as a multidisciplinary team. The Agency for Clinical Innovation (ACI) State Spinal Cord Injury Service case studies (47, 48) will be utilized during these workshops (See Supplementary Table S1). The researchers will be delivering the implementation intervention at each of the SCI services to ensure fidelity (See Supplementary Table S1). We will also check that adherence is consistent, by comparing attendance numbers between the three SCI services.

Regular training sessions for healthcare professionals working in SCI services as well as training for new staff that embeds the Psychosocial Guide into practice will be needed. Once the project is completed and the researchers are no longer on site to deliver the implementation intervention, a self-paced educational toolkit (PowerPoint presentation with narration, training videos) will be available to all healthcare professionals and students at each site as a key instructional tool to promote awareness of the Psychosocial Guide. As previously mentioned, we will also aim to provide training to key healthcare professionals who will help facilitate the workshops and will make the recommendation to re-deliver the workshops on a yearly basis. We suggest that a follow-up study could investigate the sustainment into practice, particularly to new staff and students across each site. Further to this, interventions may include in-services on psychosocial topics delivered on a regular basis, the development of a checklist of competencies for delivering person-centred care for people with SCI, and providing feedback on the experience of people living with SCI at team meetings. The interventions will be designed to educate and familiarize all healthcare professionals working in the SCI services about psychosocial care. The purpose is to optimize the implementation of the psychosocial guidelines into the practice of the SCI services (49).

### Stage 3: Evaluation

Again, guided by the organizational (inner setting) and health system level (outer setting) of the CFIR model, mixed methods assessment consisting of quantitative (online surveys) and qualitative data (interviews and focus groups) will be employed to determine the effectiveness of the interventions conducted over the 18-month period. All multidisciplinary healthcare professionals working with patients with SCI will be invited to complete a follow-up (i.e., post-intervention) survey after the delivery of the interventions. Follow-up interviews with a small number of healthcare professionals (*n* = 15) and focus groups (*n* = 20) will help to evaluate the implementation. The follow-up interviews and focus groups will explore the personal experiences of healthcare professionals as well as changes at a systems level. As mentioned previously, a small number (*n* = 10) of patients with SCI who are undertaking inpatient rehabilitation within the SCI services will also be interviewed after implementation of the Guide. The study will evaluate the success of implementing the Psychosocial Guide within the three SCI services using an audit and feedback strategy. This will be achieved through developing indicators for best psychosocial practice standards in terms of guideline uptake and adherence, health professional perceived knowledge, attitudes and skills, reduced barriers to change, changes in unit culture, and norms priorities and processes. To gauge effectiveness and level of satisfaction of care from a patient viewpoint following the introduction of the new Psychosocial Guide, a set of indicators that measures the quality of psychosocial care for SCI rehabilitation will also be determined based on patient experiences, impacts, and satisfaction with care. The outcome will establish whether there is a case for the widespread adoption of the implementation intervention in NSW and Australian hospitals to promote the use of the Guide.

### Analyses

All data will be stored on secure online data base called REDCap (41). Quantitative data will be analysed using SPSS v.25. Descriptive statistics will be generated for the items in the survey and changes over time evaluated. The software package NVIVO Version 12 (QSR International Pty Ltd, 2018) (50) will be used to analyse the qualitative data from the semi-structured interviews and focus groups. The data will be analyzed using framework analysis (e.g., CFIR). The framework method enables analysis both across and within cases, allowing for themes to be identified and interpreted, whilst still retaining the complexity of individual experiences (51). The framework method has been established as a sound method of qualitative data analysis in health service research (51, 52).

The sample size for the survey is based on the estimated number of healthcare professionals at each of the three SCI services. The pool of healthcare professionals working with people with SCI in NSW is estimated to be small compared to other health areas, which is reflective of the nature of the SCI population in NSW. Sample size for the interviews and focus groups will also be established when theoretical saturation is reached, in other words when no additional insights or concepts emerge from the data. In our experience, this is commonly achieved with 10 participants, however, we will continue to recruit participants as needed.

## Discussion

Sustaining a SCI can have a devastating lifelong impact on daily functioning, participation, and quality of life (30). Additionally, rehabilitation following SCI involves an intensive inpatient period that can be overwhelming and distressing, which in general may be for between 3 and 6 months in Australian SCI services, depending on injury severity (7, 30). This protracted time in hospital will increase the risk of isolation and institutionalization (30). Following rehabilitation, patients are discharged into the community where less support is available than when in hospital, so emphasis should be on assisting people with SCI to adjust and cope (53). Arguably, therefore, implementing psychosocial guidelines in the rehabilitation phase is essential to improve delivery of person-centred care, providing a supportive environment in which the person with SCI will learn to develop autonomy within their individual level of functioning and begin the journey of adjustment and adaptation to their injury (30, 54).

To our knowledge, this is the first study that will assess the impact of implementing psychosocial guidelines into the inpatient SCI rehabilitation setting, as well as determining potential barriers and facilitators for implementation. In addition, our study is a multifaceted endeavour that addresses the holistic needs of both people with SCI and healthcare professionals. By advancing health literacy and enhancing the capacity of healthcare professionals, it is intended to strengthen the foundations for a more inclusive, informed, and supportive healthcare landscape. Thus, it is anticipated this study will improve the delivery of psychosocial care in the acute and rehabilitation phases of SCI and therefore, provide helpful guidance on strategies that will improve implementation of psychosocial care and establish whether there is a case for the adoption of such a multidimensional implementation intervention in other SCI services in Australia and worldwide.

A possible limitation of using a survey approach is the risk of social desirability bias, particularly with this cohort. We will address this bias by delivering the survey online, making it anonymous, and by guaranteeing total confidentiality. We will also moderate the social desirability risk with information from the qualitative interviews and focus groups, where we are able to ask questions in a more general way, referencing the multidisciplinary team and the workplace systems rather than only asking about the individual's own knowledge, attitudes, and behaviours.

A number of potential challenges could arise when delivering the implementation intervention in the SCI services. For example, we anticipate some resistance to change among the healthcare professionals. To help mitigate this, high-level leaders (the Unit Directors) have endorsed the project and key informants (opinion leaders/implementation leads) from each site will be invited to co-facilitate the workshops at their site. For example, a clinical nurse educator or nurse consultant will co-facilitate the workshops for the nurses and a senior physiotherapist or clinical psychologist will co-facilitate the workshops for the allied health professionals. The healthcare professionals who express interest in co-facilitating the workshops are essentially early adopters and are in a position to include their colleagues in changing their behaviour by continuing education and demonstrating how psychosocial care can be delivered for members of the multidisciplinary team. Resource constraints may also impede the sustainability of the implementation intervention once the project has concluded, and the researchers are no longer on site to deliver the intervention. To support long-term sustainability, we plan to integrate a self-paced educational toolkit (PowerPoint with narration, training videos) into the orientation program for new students and the onboarding process for new staff.

It should be noted that the Guide has yet to be officially introduced to the SCI services and, therefore, the healthcare professionals are not expected to have knowledge of the Guide or standard training in the delivery of psychosocial care. Therefore, a more measured approach, such as a validated survey, to assessing the use of the Guide or delivery of psychosocial care was not deemed necessary as the primary focus of this implementation intervention is to launch the Guide in the SCI services. It is outside the scope of this study to develop a validated survey. This may be an area for future consideration. Furthermore, while the Guide is a suggestion for best practice for the delivery of psychosocial care to patients with SCI, these are not clinical practice guidelines and, therefore, are not meant to be mandatory but used judiciously by individuals and the team to improve person-centred care delivery. Thus, it is outside the purview of the researchers to enforce the use of the Guide in the SCI services. However, it should be noted as stated above that the Unit directors endorse the intervention to facilitate the routine implementation of the Guide within the services.

As the three sites operate independently, the researchers will make suggestions for sustainability that would enhance consistency in the promotion of the Guide across each site post-intervention. However, again it is outside the purview of the researchers to mandate this. It is also outside the scope of this study to explore the long-term impact post-intervention. Future research could investigate the sustainability and long-term impact of the implementation intervention at each of the three SCI services. It is hoped that by investigating possible benefits of injecting a psychosocial guide into the three SCI services this will result in improve care and rehabilitation outcomes.

## Data Availability

The original contributions presented in the study are included in the article/Supplementary Material, further inquiries can be directed to the corresponding author.
