# Supplementary material for: Implementing psychosocial guidelines into specialized spinal cord injury rehabilitation services to strengthen person-centred health care: protocol for a mixed methods study
Source: Front Rehabil Sci. 2025 Apr 7;6:1537890. doi: 10.3389/fresc.2025.1537890 (PMC12009950; doi:10.3389/fresc.2025.1537890)
Supplement: Supplementary file 1 [file Table1.pdf]

## Standards for Reporting Implementation Studies: the StaRI checklist for completion

The StaRI standard should be referenced as: Pinnock H, Barwick M, Carpenter C, Eldridge S, Grandes G, Griffiths CJ, Rycroft-Malone J, Meissner P, Murray E, Patel A, Sheikh A, Taylor SJC for the StaRI Group. Standards for Reporting Implementation Studies (StaRI) statement. *BMJ* 2017;356:i6795

The detailed Explanation and Elaboration document, which provides the rationale and exemplar text for all these items is: Pinnock H, Barwick M, Carpenter C, Eldridge S, Grandes G, Griffiths C, Rycroft-Malone J, Meissner P, Murray E, Patel A, Sheikh A, Taylor S, for the StaRI group. Standards for Reporting Implementation Studies (StaRI). Explanation and Elaboration document. *BMJ Open* 2017 2017;7:e013318

Notes: A key concept of the StaRI standards is the dual strands of describing, on the one hand, the implementation strategy and, on the other, the clinical, healthcare, or public health intervention that is being implemented. These strands are represented as two columns in the checklist.

| | |
|---|---|
| The primary focus of implementation science is the implementation strategy (column 1) and the expectation is that this will always be completed. | The evidence about the impact of the intervention on the targeted population should always be considered (column 2) and either health outcomes reported or robust evidence cited to support a known beneficial effect of the intervention on the health of individuals or populations. |

The StaRI standards refers to the broad range of study designs employed in implementation science. Authors should refer to other reporting standards for advice on reporting specific methodological features. Conversely, whilst all items are worthy of consideration, not all items will be applicable to, or feasible within every study.

| Checklist item | | Implementation Strategy | Intervention |
|---|---|---|---|
| | | "Implementation strategy" refers to how the intervention was implemented | "Intervention" refers to the healthcare or public health intervention that is being implemented. |
| **Title and abstract** | | | |
| Title *(Identification as an implementation study, and description of the methodology in the title and/or keywords)* | 1 | Implementing psychosocial guidelines into specialized SCI rehabilitation services to strengthen person-centred health care: protocol for a mixed methods study | |
| Abstract *(Identification as an implementation study, including a description of the implementation strategy to be tested, the* | 2 | In Australia, a Psychosocial Guide was developed for multidisciplinary healthcare staff working in the rehabilitation of people with a spinal cord injury (SCI) but was never thoroughly integrated into the SCI rehabilitation process. Recognizing the difficulties of integrating past versions of the Psychosocial Guide into SCI rehabilitation practice, the primary aim of this study is to implement and integrate the latest Guide (upgraded in July 2023) into the three specialist SCI services in NSW, Australia. | |

| | | | |
|---|---|---|---|
| *evidence-based intervention being implemented, and defining the key implementation and health outcomes.)* | | A mixed-methods approach (surveys, interviews, and focus groups) will aid in the development of an implementation intervention employing educational, structural, and nudge (behavioural change) strategies that will be implemented over a period of 18-months to facilitate integration of the Psychosocial Guide into SCI services in NSW, Australia.<br><br>Post-intervention follow-up (surveys, interviews, and focus groups) with healthcare workers will identify facilitators and barriers to implementing the Psychosocial Guide. Success of implementation will be determined by analyzing any shifts in perceived knowledge, attitudes, and behaviour of staff and cultural/structural processes observed through comparing baseline and post-intervention qualitative and quantitative data. To capture lived experience insight, 15 people with SCI will be interviewed. | |
| **Introduction** | | | |
| Introduction<br>*(Description of the problem, challenge or deficiency in healthcare or public health that the intervention being implemented aims to address.)* | 3 | SCI (SCI) results from a sudden traumatic event or non-traumatic disease/disorder, disrupting multiple aspects of a person's life, including physical functioning, health, emotional wellbeing, relationships, and social participation. Rehabilitation following SCI requires an integrated, evidence-based and person-centred approach by an expert multidisciplinary team. If ppsychosocial aspects, including cognitive issues, are overlooked or underestimated, the person with SCI is likely to have poorer adjustment and may experience negative outcomes, such as anxiety and depression, difficulties with self-management, and rehospitalisation due to complications (e.g., a pressure injury). In Australia, a Psychosocial Guide was consequently developed for healthcare staff working in SCI rehabilitation but was never thoroughly integrated into the SCI rehabilitation process. Recognizing the difficulties of integrating past versions of the Psychosocial Guide into SCI rehabilitation practice, the primary aim of this study is to implement and integrate the latest Guide (upgraded in July 2023 to include cognitive impairment guidelines) into the three specialist SCI services in NSW, Australia. | |
| Rationale | 4 | *(The scientific background and rationale for the implementation strategy [including any underpinning theory/framework/model, how it is expected to achieve its effects and any pilot work].)*<br><br><u>Rationale for the implementation strategy</u><br>The provision of high-quality, holistic, person-centred care for the complex condition of SCI entails bridging the gap between evidence-based psychosocial guidelines and day-to-day clinical practice. The Consolidated Implementation Research Framework will be used to | *{The scientific background and rationale for the intervention being implemented [including evidence about its effectiveness and how it is expected to achieve its effects].)*<br><br><u>Rationale for the intervention</u><br>The biopsychosocial model and International Classification for Functioning, Disability, and Health framework (body function and structure, activity, and participation) underpins the delivery of rehabilitation, recognizing the complex interaction |

| | | | |
|---|---|---|---|
| | | understand the context and practice drivers when developing the implementation strategy. | between the person's health condition, their environment, and personal factors. |
| Aims and objectives<br>(*The aims of the study, differentiating between implementation objectives and any intervention objectives.)* | 5 | Intervention objective: the primary aim of this study is to implement and integrate the latest Psychosocial Guide (upgraded in July 2023) into the three specialist SCI services in NSW, Australia.<br><br>Implementation objectives: (i) to explore individual, team, organizational (inner setting), and health system level (outer setting) barriers and facilitators to the implementation of the Guide using the Consolidated Framework for Implementation Research (CFIR); (ii) to increase awareness, knowledge, and understanding of the Psychosocial Guide; and (iii) to co-design a set of implementation strategies and embed a plan to promote sustainability of the intervention in the SCI services. | |
| **Methods: description** | | | |
| Design<br>*(The design and key features of the evaluation, [cross referencing to any appropriate methodology reporting standards] and any changes to study protocol, with reasons)* | 6 | A mixed methods assessment consisting of quantitative (online surveys) and qualitative data (interviews and focus groups) will be employed for evaluation. First, we will assess the context for implementation through surveying a range of healthcare professionals (HCPs) to gauge the organisational commitment and readiness within three different SCI services to implement a more systematic application of the Psychosocial Guide. Key informant interviews and focus groups will advise on the current climate of the SCI services, engage key HCPs as champions to help promote guideline uptake and adherence, and help to elicit potential strategies for the implementation intervention. Baseline interviews with people with a SCI (n=15) will document experiences of psychosocial care delivery in the three SCI services.<br><br>Themes and information derived from baseline data will guide the development of a range of active implementation strategies supporting the application of the Psychosocial Guide that will help to embed the Guide into clinical practice and institutionalise cultural changes.<br><br>Guided by the organizational (inner setting) and health system level (outer setting) of the CFIR model, mixed methods assessment consisting of quantitative (online surveys) and qualitative data (interviews and focus groups) will be employed to determine the effectiveness of the interventions conducted over the 18-month period. All multidisciplinary staff will be invited to complete a follow-up (i.e., post-intervention) survey after the delivery of the interventions. Follow-up interviews with a small number of HCPs (n=15) and focus groups (n=20) will help to evaluate the implementation outcomes. The follow-up interviews and focus groups will explore the personal experiences of HCPs as well as changes at a systems level. A small number (n=15) of adults with SCI who have undertaken inpatient rehabilitation within the services will also be interviewed after implementation of the guidelines. | |

| | | | |
|---|---|---|---|
| Context<br>*The context in which the intervention was implemented. [Consider social, economic, policy, healthcare, organisational barriers and facilitators that might influence implementation elsewhere].)* | 7 | The three participating specialist SCI services are part of a statewide service in NSW, Australia, delivering inpatient care to people with SCI at different stages of post-injury management (i.e., acute and/or rehabilitation), requiring contextual understanding for consistency and customization of intervention between settings. Understanding the baseline context, including awareness and knowledge of the Guide through survey and in-depth interviews, in addition to focus groups to understand the perspectives of HCPs and how confident they are in delivering psychosocial care will provide an individual and multidisciplinary working team context. The Consolidated Framework for Implementation Research (CFIR) provides an ideal structure with which to explore organizational (e.g., culture, leadership, communications, perceived need for change, resources) and health system level (e.g., values and beliefs, policies, partnerships with consumer organisations) barriers and facilitators to the implementation of the Guide. This will be achieved through interviews and focus groups with the multidisciplinary healthcare workers in SCI services and individuals living with SCI, according to CFIR domains. | |
| Targeted 'sites' | 8 | *(The characteristics of the targeted 'site(s)' [e.g., locations/personnel/resources etc.] for implementation and any eligibility criteria.)*<br><br>The study will be conducted in three specialist SCI services in Sydney, Australia, with one facility mostly providing acute care, another only subacute rehabilitation and a third Unit providing both acute care and subacute rehabilitation. The three SCI services have variations in catchment areas, as well as infrastructure and organisation of professional teams, workflows, and processes for SCI rehabilitation. | *(The population targeted by the intervention and any eligibility criteria.)*<br><br>HCPs including nurses, physiotherapists, occupational therapists, speech pathologists, medical specialists, social workers, psychologists, and psychiatrists working in the three SCI services will be invited to participate.<br><br>Inclusion criteria will consist of a) health care workers from each of the three SCI services located at three NSW hospitals who volunteer to participate in the study; b) a minimum of 12-months experience; c) representation by all disciplines, and d) being able to communicate effectively in English. The researchers will invite key informants that meet the inclusion criteria to be interviewed to ensure we capture the views of a broad range of HCPs, including opinion leaders and managers. |
| Description | 9 | *(A description of the implementation strategy)*<br><br>This intervention will be comprised of educational and practical workshops, supported by structural/procedural changes and behavioural change (nudge) strategies. The in-person workshops will be held at each of the three SCI services. The educational workshops will be delivered to | *(A description of the intervention)*<br><br>A collaborative, team-based approach in contributing to individualised rehabilitation plans through communication, emotional support, understanding adjustment and coping, and developing strategies for supporting the person with SCI in |

| | | | |
|---|---|---|---|
| | | the nurses and allied health groups separately at each of the three sites. The practical workshops will be delivered to the multidisciplinary team of health professionals at each site.<br><br>Following the completion of the study, a self-paced educational toolkit will be made available online to all staff, specifically for the induction of new staff and students.<br><br>Nudges will also be employed throughout the intervention time, involving various environmental cueing/priming strategies at each site (e.g., infographics displayed on notice boards in the three sites). | learning new skills for independent functioning everyday activities.<br><br><u>Educational workshop</u><br>The researchers will develop a PowerPoint presentation as a key instructional tool to enhance the delivery of educational content aimed to introduce the Guide to the HCPs and provide a basic overview of the purpose and content of the Guide.<br><br><u>Practical workshop</u><br>The focus of these workshops will be case studies depicting a SCI patient in either acute or rehabilitation care. Role plays, practice enabling/modelling, interactive strategies in line with adult learning principles.<br>The case studies can be accessed on the ACI website: (acute: http://aci.health.nsw.gov.au/__data/assets/pdf_file/0011/166619/ACI-SSCIS-psychosocial-strategy-case-study1.pdf; rehabilitation: http://aci.health.nsw.gov.au/__data/assets/pdf_file/0003/166620/ACI-SSCIS-psychosocial-strategy-case-study2.pdf)<br><br><u>Nudges</u><br>3 x infographics and 1 x screensaver will be developed by the researchers and made available/distributed throughout the SCI services to be displayed on the noticeboards and on the computers in the SCI services sites respectively.<br>Case studies with reflection questions will be circulated via email to give people the opportunity to apply the Guide to scenarios similar to those they encounter on a daily basis.<br>Psychosocial stories and feedback (feedback survey) from patients will also be circulated via email to provide feedback |

| | | | |
|---|---|---|---|
| | | | on the experience of people living with SCI and their family members.<br><br><u>Self-paced educational toolkit</u><br>A PowerPoint presentation with narration will be available to all staff and students at each site as a key instructional tool to promote awareness of the Psychosocial Guide.<br><br><u>Psychosocial working group</u><br>Establish a working group with psychosocial 'champions' across the three sites to promote the Guide and continue learning on psychosocial care during and post intervention. This group will also facilitate implementation of other structural or procedural strategies to improve interdisciplinary communication mechanisms, decision-making and planning processes or address factors affecting adoption identified by the CFIR. |
| Sub-groups<br>*(Any sub-groups recruited for additional research tasks, and/or nested studies are described)* | 10 | As care recipients, up to 15 adults aged between 18-80 years with SCI, able to communicate effectively in English, who are currently undergoing rehabilitation, and not experiencing severe mental illness or at risk-behaviours will also be interviewed pre- and post-rehabilitation, to obtain their perceptions and awareness of psychosocial care during rehabilitation. | |
| **Methods: evaluation** | | | |
| Outcomes | 11 | *(Defined pre-specified primary and other outcome(s) of the implementation strategy, and how they were assessed. Document any pre-determined targets)*<br><br>Psychosocial care is a part of every team member's role and requires all HCPs to be aware of these factors, knowledgeable, and capable in providing evidence-based, person-centred SCI rehabilitation. Awareness of the Guide, practice development, penetration and sustainability in using the Guide are outcomes of the implementation strategy. | *(Defined pre-specified primary and other outcome(s) of the intervention [if assessed], and how they were assessed. Document any pre-determined targets)*<br><br>The study will evaluate the success of implementing the Psychosocial Guide within the three SCI services using an audit and feedback strategy. This will be achieved through developing indicators for best psychosocial practice standards in terms of guideline uptake and adherence, health professional perceived knowledge, attitudes/intentions and skills, reduced barriers to change, changes in unit culture, and |

| | | | |
|---|---|---|---|
| | | The use of the Consolidated Framework for Implementation Research (CFIR) will provide a structure with which to explore organizational and health system level barriers and facilitators to the implementation of the Guide. These barriers and facilitators to implementation will be assessed using qualitative interviews and focus groups with HCPs professionals at each of the three sites. | norms, priorities and processes. To gauge effectiveness and level of satisfaction of care from a patient viewpoint following the introduction of the new Psychosocial Guide, a set of indicators that measures the quality of psychosocial care for SCI rehabilitation will also be determined based on patient experiences, impacts, and satisfaction with care. |
| Process evaluation<br>*(Process evaluation objectives and outcomes related to the mechanism by which the strategy is expected to work)* | 12 | Process evaluation will target proposed mechanisms of action, such as increasing awareness and understanding of psychosocial issues, upskilling and supporting multidisciplinary team with strategies, and providing influence/leadership through local champions. Rates of workshop attendance and numbers of HCPs visiting online education workshop material will be recorded. Outcome measures will include increased awareness and knowledge of the Guide, information that intervention is acceptable, appropriate and feasible for local use, adoption by clinicians (i.e., percent of health professionals who complete training and express behavioural intention) and adoption by key decision-makers and implementers (i.e., making practice and policy changes). | |
| Economic evaluation | 13 | *(Methods for resource use, costs, economic outcomes and analysis for the implementation strategy)*<br><br>Not applicable for this study | *(Methods for resource use, costs, economic outcomes and analysis for the intervention)*<br><br>Not applicable for this study |
| Sample size<br>*(Rationale for sample sizes [including sample size calculations, budgetary constraints, practical considerations, data saturation, as appropriate])* | 14 | The sample size for the survey is based on the estimated number of HCPs at each of the three sites. The pool of HCPs working with people with SCI in NSW is estimated to be small compared to other health areas, which is reflective of the nature of the SCI population in NSW. Sample size for the interviews and focus groups will also be established when theoretical saturation is reached, in other words when no additional insights or concepts emerge from the data. In our experience, this is commonly achieved with 10 participants, however, we will continue to recruit participants as needed. Practical considerations include availability of HCPs, as well as availability of patients with SCI undergoing rehabilitation to participate in surveys, interviews/focus groups to understand the attitudes, beliefs and experiences. Scope of project limited by time for sustainment of approaches to ensure HCPs are aware of the Guide and to consider the Guide overtime. | |
| Analysis<br>*(Methods of analysis [with reasons for that choice])* | 15 | Descriptive statistics will be generated for the survey items and changes over time evaluated. Qualitative data from the baseline and follow-up semi-structured interviews and focus groups will be analyzed using framework analysis (e.g., CFIR). The framework method enables analysis both across and within cases, allowing for themes to be identified and interpreted, whilst still retaining the complexity of individual experiences. Pre-and post-intervention changes in the beliefs | |

| | | |
|---|---|---|
| | | and attitudes of HCPs will be compared. The framework method has been established as a sound method of qualitative data analysis in health service research. |
| Sub-group analyses *(Any a priori sub-group analyses [e.g., between different sites in a multicentre study, different clinical or demographic populations], and sub-groups recruited to specific nested research tasks)* | 16 | Staffing models and other structural differences (such as meeting frequency and types, goal planning activities, communication and engagement) at each site will be compared to provide a basic overview of the different clinical populations at each site. |

| **Results** | | | |
|---|---|---|---|
| Characteristics | 17 | *(Proportion recruited and characteristics of the recipient population for the implementation strategy)* The implementation intervention will be launched mid-2025. | *(Proportion recruited and characteristics [if appropriate] of the recipient population for the intervention)* Not applicable. The implementation intervention will be launched mid-2025. |
| Outcomes | 18 | *(Primary and other outcome(s) of the implementation strategy)* The implementation intervention will be launched mid-2025. | *(Primary and other outcome(s) of the Intervention (if assessed)* Not applicable. The implementation intervention will be launched mid-2025. |
| Process outcomes *(Process data related to the implementation strategy mapped to the mechanism by which the strategy is expected to work)* | 19 | The implementation intervention will be launched mid-2025. | |

| | | | |
|---|---|---|---|
| Economic evaluation | 20 | *(Resource use, costs, economic outcomes and analysis for the implementation strategy)*<br><br>Not applicable | *(Resource use, costs, economic outcomes and analysis for the intervention)*<br><br>Not applicable |
| Sub-group analyses<br>*(Representativeness and outcomes of subgroups including those recruited to specific research tasks)* | 21 | Recruitment of patients with SCI who are actively experiencing their patient journey to ensure that their perceptions are recent and current as compared to people living with SCI who are living in the community and may not recall events from hospital. The qualitative interviews of patients with SCI are included to provide invaluable perspectives as recipients of rehabilitation that includes psychosocial care in their encounters of spinal service delivery. They will be able to share their experiences of the behaviours of HCPs and will give a snapshot in time. | |
| Fidelity/ adaptation | 22 | *(Fidelity to implementation strategy as planned and adaptation to suit context and preferences)*<br><br>The researchers will be delivering the implementation intervention at each of the SCI services to ensure fidelity. Post-intervention, all staff and students from each site will be able to access a self-paced educational toolkit (PowerPoint presentation with narration) as a key instructional tool to promote awareness of the Psychosocial Guide during and post intervention. | *(Fidelity to delivering the core components of intervention [where measured])*<br><br>The researchers will be delivering the implementation intervention at each of the SCI services to ensure fidelity. We will also check that adherence is consistent, by comparing attendance numbers between the three SCI services. |
| Contextual changes<br>*(Contextual changes [if any] which may have affected outcomes)* | 23 | The implementation intervention will be launched mid-2025. | |
| Harms<br>*(All important harms or unintended* | 24 | Unintended effects may result from HCPs not being able to access training to increase knowledge and skills in areas suggested by the Guide, such as mental health first aid. | |

<table>
<tr><td>effects in each group)</td><td></td><td colspan="2"></td></tr>
<tr><td colspan="4">Discussion</td></tr>
<tr><td>Structured discussion<br>(Summary of findings, strengths and limitations, comparisons with other studies, conclusions and implications)</td><td>25</td><td colspan="2">As this is a protocol paper, we are unable to provide a summary of findings at this stage.</td></tr>
<tr><td>Implications</td><td>26</td><td>(Discussion of policy, practice and/or research implications of the implementation strategy [specifically including scalability])<br><br>A self-paced educational toolkit in the form of a PowerPoint presentation with narration will be available to all staff and students at each site as a key instructional tool to promote awareness of the Psychosocial Guide during and post intervention. This will enable all staff (including night shift and weekend staff) and students to access the educational material utilised during the implementation intervention, not just HCPs that were available during the scheduled intervention activities. Organisational leadership and support will ensure the Guide becomes embedded into practice, professional development, and training.</td><td>(Discussion of policy, practice and/or research implications of the intervention [specifically including sustainability])<br><br>A self-paced educational toolkit in the form of a PowerPoint presentation with narration will be available to all staff and students at each site as a key instructional tool to promote awareness of the Psychosocial Guide during and post intervention.<br><br>An adjunct strategy will be to build and foster a guiding coalition (community of practice within the SCI services) with designated “champions”, who can model, support, and influence staff behaviours.</td></tr>
<tr><td colspan="4">General</td></tr>
<tr><td>Statements<br>Include statement(s) on regulatory approvals</td><td>27</td><td colspan="2">The project received funding from icare NSW (AUD $231,314) over three years. This study is funded by icare NSW (Innovation and Partnerships). This funding source had no role in the design of this study. Nor will they have any role during its execution, data collection or analyses, interpretation of the data, writing the manuscript, or decision to submit results.</td></tr>
</table>

| | | |
|---|---|---|
| *[including, as appropriate, ethical approval, confidential use of routine data, governance approval], trial/study registration [availability of protocol], funding and conflicts of interest)* | | Ethics approval was granted by the Northern Sydney Local Health District (NSLHD) Human Research Ethics Committee (HREC) (2022/ETH02224) on 20 Feb 2023. Written consent to participate will be obtained from all participants prior to study commencement using a NSLHD HREC approved consent form.<br><br>This study has been retrospectively registered with the Australian New Zealand Clinical Trials Registry on the 7$^{th}$ of May 2024. The registration number is: ACTRN12624000581561<br><br>One of the researchers (CM) have part of their salary paid from the research funding received for this project. This study also forms part of a PhD candidature for one of the researchers (AMS). |
